# Little-known complication: acute type B aortic dissection during transcatheter edge-to-edge repair for mitral regurgitation

**DOI:** 10.1093/ehjcr/ytaf091

**Published:** 2025-02-25

**Authors:** Fumiya Chubachi, Arudo Hiraoka, Takao Morikawa

**Affiliations:** Department of Cardiovascular Surgery, The Sakakibara Heart Institute of Okayama, 2-5-1 Nakaicho, Kita-ku, Okayama 700-0804, Japan; Department of Cardiovascular Surgery, The Sakakibara Heart Institute of Okayama, 2-5-1 Nakaicho, Kita-ku, Okayama 700-0804, Japan; Department of Cardiovascular Surgery, The Sakakibara Heart Institute of Okayama, 2-5-1 Nakaicho, Kita-ku, Okayama 700-0804, Japan

We previously reported two cases of acute type B aortic dissection (ATBAD) during transcatheter edge-to-edge repair (TEER) by MitraClip (Abbott Vascular). Recently, ATBAD was observed in another case, a 92-year-old woman treated with TEER by the PASCAL system (Edwards Lifesciences, Irvine, CA, USA). The entry was located in the middle oesophagus near the left atrium in all three patients. Thin-slice computed tomography revealed that the entry was connected to the intercostal artery (*Panel A*). This finding suggests that ATBAD was caused by an intimal injury due to a pulled-out intercostal artery, rather than direct aortic injury, in all three cases. An intraoperative transesophageal echocardiography (TEE) probe may retract the descending aorta, fixed by the intercostal artery, and push out the intima of the intercostal artery (*Panel B*). The injury of intima is a new entry and main mechanism of ATBAD during TEER. The direction of the TEE probe is towards the aorta at the retroflex position in the middle oesophagus; the retroflex position is frequently used to obtain a clear view of the mitral valve in TEER (*Panel C*). On the other hand, the anteflex position is commonly used in the Watchman procedure; therefore, the risk of ATBAD is estimated to be higher in TEER (*Panel D*). Regarding predictors, a limited working space of the TEE probe (middle oesophagus surrounded by the spine, descending aorta and left atrium) was considered as a risk of ATBAD. Additionally, the intercostal artery originating from the same level of the descending aorta is thought to be a strong predictor of ATBAD during TEER.

**Figure:**
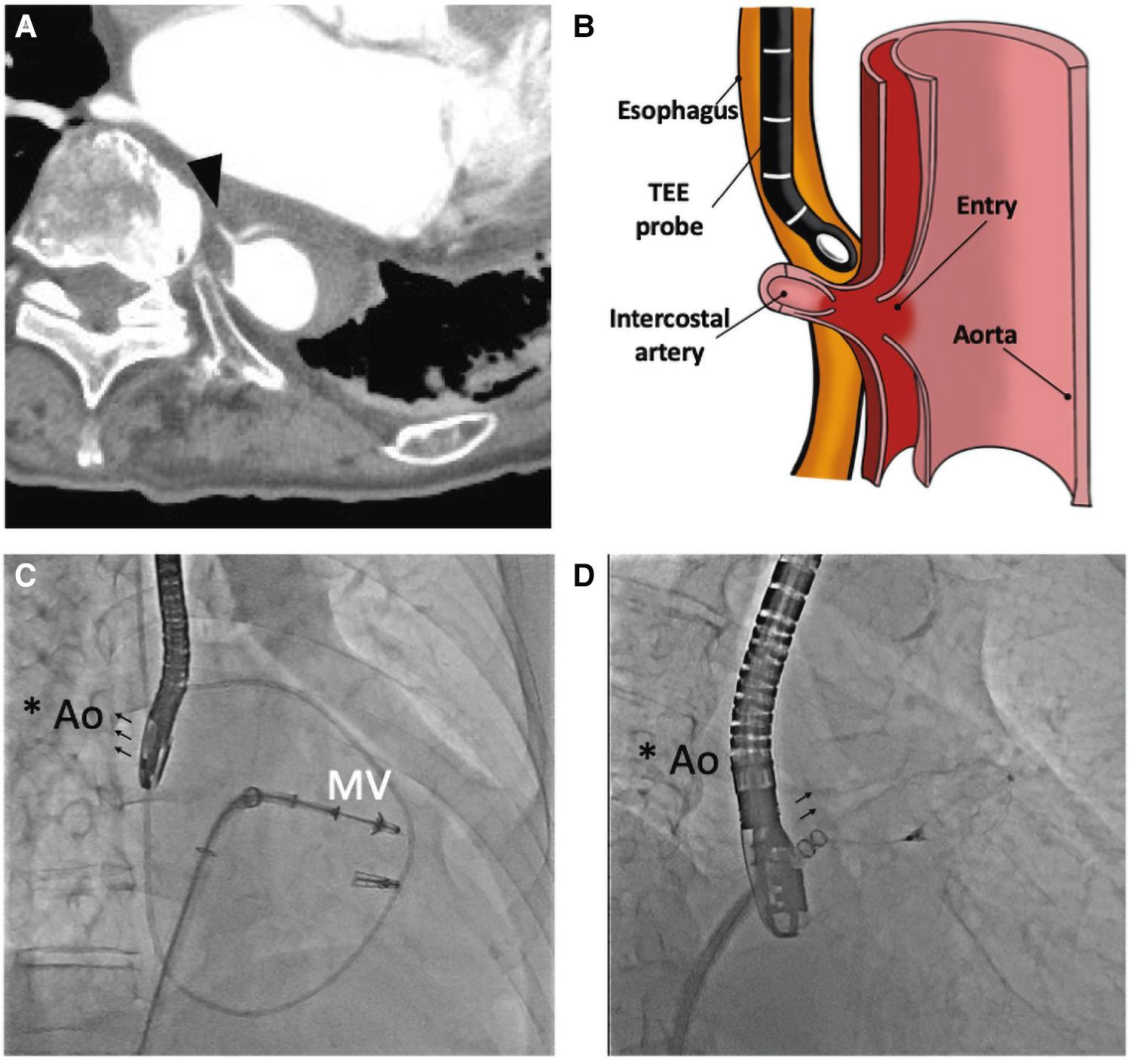
Computed tomography (*A*) and schema (*B*) of the mechanism of aortic dissection during transcatheter edge-to-edge repairTEER. Difference of the direction of the transoesophageal echocardiographyTEE probe (*C* and *D*).

**Consent:** Written consents for submission and publication of this case report including images were obtained from the patients in line with the COPE guidelines.

**Funding:** None declared.

## Data Availability

The data underlying this article will be shared on reasonable request to the corresponding author.

